# Prognostic Significance of Peripheral Artery Disease in Patients with Acute Coronary Syndrome Undergoing Percutaneous Coronary Intervention

**DOI:** 10.31083/j.rcm2411332

**Published:** 2023-11-24

**Authors:** Yihua Xia, Kangning Han, Yujing Cheng, Zhijian Wang, Fei Gao, Xiaoteng Ma, Yujie Zhou

**Affiliations:** ^1^Beijing Institute of Heart Lung and Blood Vessel Disease, 100029 Beijing, China; ^2^Department of Cardiology, Beijing Anzhen Hospital, Capital Medical University, 100029 Beijing, China

**Keywords:** peripheral artery disease, acute coronary syndrome, percutaneous coronary intervention, cardiovascular outcomes

## Abstract

**Background:**

Peripheral artery disease (PAD) elevates the risk of adverse outcomes. The current work aimed to evaluate the influence of PAD in acute coronary syndrome (ACS) cases administered percutaneous coronary intervention (PCI), and to determine whether PAD adds incremental prognostic value to the global registry of acute coronary events (GRACE) scale.

**Methods:**

To retrospectively analyze a single-center, prospective cohort trial, we consecutively included ACS cases administered PCI. Individuals with and without PAD were comparatively examined for clinical outcomes. The primary endpoint was major adverse cardiovascular events (MACEs), a compound item encompassing all-cause death, myocardial infarction (MI), stroke and repeat revascularization. The added value of PAD based on a reference model was examined.

**Results:**

PAD was detected in 179 (10.4%) of the 1,770 included patients. The incidence rates of MACEs (40.3% vs. 17.9%), all-cause death (11.2% vs. 1.6%), cardiovascular death (8.9% vs. 1.4%), MI (8.4% vs. 2.2%) and repeat revascularization (30.2% vs. 15.2%) were all markedly elevated in PAD cases in comparison with the non-PAD group (*p* < 0.001). After adjusting for other confounding variates, PAD independently predicted MACE occurrence (hazard ratio = 1.735, 95% confidence interval: 1.281–2.351). Addition of PAD resulted in remarkably increased predictive performance for MACE compared to the baseline GRACE score (Harrell’s C-statistic: 0.610 vs. 0.587, *p* < 0.001; net reclassification improvement: 0.134, *p* < 0.001; integrated discrimination improvement: 0.035, *p* < 0.001).

**Conclusions:**

In ACS cases administered PCI, PAD independently worsens clinical outcomes and adds incremental value to the GRACE risk score.

## 1. Introduction

Acute coronary syndrome (ACS) constitutes an important public health challenge that imposes an economic burden worldwide, causing almost 50% of deaths related to coronary heart disease (CHD) [1]. Accurately stratifying risk is of great value in the clinical treatment of acute and chronic CHD. Nevertheless, current predictive models for identifying potential poor prognosis in patients with ACS, including the global registry of acute coronary events (GRACE) scoring scale, do not consider coronary artery and noncoronary lesions.

Peripheral arterial disease (PAD) represents a non-coronary sign of atherosclerosis, which often coexists with coronary artery disease (CAD) and cerebrovascular disease [2, 3]. More than 40% CAD cases also show concurrent PAD, imposing an important burden of cardiovascular events and increasing overall mortality [4]. Individuals with combined CAD and PAD administered coronary revascularization show elevated rates of perioperative and long-term complications in comparison with patients without PAD [5]. Studies have confirmed that PAD cases administered percutaneous coronary intervention (PCI) have an elevated rate of major adverse cardiovascular events (MACEs) in comparison with non-PAD cases, with PAD independently and significantly predicting death [6, 7].

However, the prognostic potential of PAD in ACS cases administered PCI is unknown. Additionally, reports evaluating whether PAD addition improves the GRACE scale are scarce. Therefore, this work aimed to determine the potential of PAD in predicting prognosis in patients with ACS following PCI, and to investigate whether PAD improves the predictive potential of the GRACE risk scoring system.

## 2. Materials and Methods

### 2.1 Populations

The current retrospective analysis examined a single-center, prospective cohort trial (ChiCTR1800017417) that consecutively included 1770 ACS cases with elective PCI between June 2016 and November 2017 in Beijing Anzhen Hospital, Capital Medical University. Diagnostic criteria for ACS followed the current guidelines [defined as unstable angina (UA), non-ST-segment elevation myocardial infarction (NSTEMI) and ST-segment elevation myocardial infarction (STEMI)] [8]. Exclusion criteria were: (1) age <18 years; (2) no or incomplete data; (3) previously administered coronary artery bypass grafting (CABG); (4) known cancer history. Ultimately, totally 1726 individuals were included. The study followed the Declaration of Helsinki on Human Research, with approval from the Institutional Review Board of Beijing Anzhen Hospital, Capital Medical University.

### 2.2 Data Collection

Demographic data and medical and medication histories were obtained with a standard questionnaire. Upon admission, blood pressure assessment was carried out. Then, albumin (ALB), lipid levels, fasting plasma glucose (FPG), glycosylated hemoglobin, high-sensitivity C-reactive protein (hs-CRP) and creatinine amounts in initial fasting blood specimens during hospitalization, collected upon 12 h of fasting, were assessed by the central laboratory of Beijing Anzhen Hospital. Diagnosis of PAD was based on ultrasound results, symptoms or past history, including decreased or no pulsation, vascular revascularization on aorta or extremities, exercise-associated continuous claudication, extremity ischemic rest pain, amputation due to extremity ischemia, confirmed aortic aneurysm, or confirmed renal artery stenosis. Ultrasound scans were not routinely screened for cases without any symptoms or a past history of the disease (**Supplementary Table 1**). Ultrasound-based criteria for PAD were non-coronary aortic and arterial-associated vascular diseases, and lumen stenosis beyond 50%. Patients with systolic (SBP) and/or diastolic (DBP) blood pressure levels of 140 and 90 mmHg or higher, respectively, in measurements performed on distinct days or being administered anti-hypertensive drugs were deemed to have hypertension. Type 2 diabetes mellitus (T2DM) was diagnosed with blood glucose content ≥11.1 mM, FBG ≥7.0 mM, 2-hour blood glucose upon oral glucose tolerance test ≥11.1 mM and/or or treatment with hypoglycemic products. Dyslipidemia was diagnosed as fasting total cholesterol (TC) content >200 mg/dL, low-density lipoprotein cholesterol (LDL-C) content >130 mg/dL, triglyceride (TG) content >150 mg/dL, high-density lipoprotein cholesterol (HDL-C) content <40 mg/dL and/or prolonged use of lipid-lowering drugs. GRACE risk scores were determined for patients.

### 2.3 Follow-Up and Study Endpoints

Upon discharge, follow-up was carried out at 1, 6, 12, 18, 24, 30 and 36 months, collecting data on adverse events from patient records and by phone by 3 experienced staff unaware of baseline features. Patients were followed up at 1, 6, 12, 18, 24, 30 and 36 months after discharge. Information on all adverse events (primary compound endpoint) was obtained by telephone contact with patients or their family members and was determined by careful review of the corresponding medical records by trained professional follow-up staff who were unaware of the baseline characteristics of the patients followed.

The initial patient was enrolled in June 2016, and the final follow-up occurred in December 2019. The primary compound endpoint was MACEs, encompassing all-cause death, stroke, myocardial infarction (MI) and repeat revascularization. Stroke was reflected by symptoms of neurological injury from ischemic lesions detected by computed tomography and/or magnetic resonance imaging. MI was characterized by increased cardiac troponin and/or creatine kinase amounts based on respective reference values, signs of ischemia and/or electrocardiographic data indicating myocardial ischemia. Unplanned repeat revascularization was reflected by recurrent or persistent ischemic symptoms leading to vessel revascularization.

### 2.4 Statistical Analysis

Based on PAD status, the patients were assigned to two groups. Continuous data with normal distribution are mean ± standard deviation, and were assessed for differences by two-sample *t*-test; those with non-normal distribution were presented as median and interquartile range and assessed for differences by the Mann-Whitney U test. Categorical data were represented by number (percentage) and assessed for differences by the χ^2^ test or Fisher’s exact test. The Kaplan–Meier method was utilized to assess event rates during follow-up, and group-differences were assessed by the log rank test. Multivariate Cox proportional hazard regression was utilized to adjust for confounding factors, generating hazard ratios (HRs) and 95% confidence intervals (CIs) for PAD in MACEs. Variables that were identified as potential risk factors for the primary study endpoint in the univariate Cox proportional risk model analysis (*p* < 0.05) or were considered to be potentially clinically significant in clinical practice were included in the multifactor Cox proportional risk model analysis. Also, variables that may have been covariates with PAD were excluded from the study. The variables included in the multifactor Cox proportional risk model included: age, gender, body mass index (BMI), family history of CAD, current smokers, hypertension, diabetes, hyperlipidemia, and previous MI, previous PCI, heart failure, heart rate, SBP, DBP, Killip classification, cardiac arrest, lymphocyte count, neutrophil count, monocyte count, LDL-C, hs-CRP, glycosylated hemoglobin, left ventricular ejection fraction (LVEF), proximal left anterior descending artery (LAD) stenosis, SYNTAX score, complete revascularization, angiotensin-converting enzyme inhibitor (ACEI) or angiotensin receptor blocker (ARB), aspirin, β-blockers, and insulin uses at discharge. The interaction effect was assessed by the likelihood ratio test. Harrell’s C-statistics, net reclassification improvement (NRI) and integrated discrimination improvement (IDI) were calculated for assessing the added value of PAD on the capabilities in predicting MACEs. Data analysis utilized SPSS 26.0 (IBM Corp., Armonk, NY, USA) and R3.6.3 (R Foundation for Statistical Computing, Vienna, Austria), with two-tailed *p* < 0.05 suggesting statistical significance.

## 3. Results

### 3.1 Baseline Patient Features

Totally 1726 cases were analyzed, comprising 179 (10.4%) patients who were identified with PAD at baseline, of which 76.7% (n = 1547) were male. The patients were 60 ± 10 years. Patient baseline features are shown in Table 1. PAD cases were older (66 ± 10), had higher odds of being male and more often had hypertension, diabetes, heart failure, prior myocardial infarction, previous PCI, a family history of CAD, and a lower rate of smoking. Regarding laboratory examinations, participants with PAD had higher levels of hs-CRP, FPG and glycosylated hemoglobin, and decreased left ventricular ejection fraction. Based on angiographic data, individuals with PAD had elevated SYNTAX (synergy between percutaneous coronary intervention with taxus and cardiac surgery) scores and were less likely to achieve complete revascularization. In terms of discharge medications, PAD cases were more often administered ACEIs or angiotensin receptor blockers (ARBs), but were less likely to be administered aspirin or statins.

**Table 1.** **Baseline characteristics of patients with and without PAD**.

		With PAD (n = 179)	Without PAD (n = 1547)	*p* value
Age, years		66 ± 10	59 ± 10	<0.001
Gender, male, n (%)		148 (82.7)	1175 (88.8)	0.440
BMI, kg/$m^{2}$		25.6 ± 3.0	25.7 ± 3.1	0.857
Heart rate, bpm		71 ± 11	68 ± 9	<0.001
SBP, mmHg		132 ± 18	130 ± 16	<0.001
DBP, mmHg		72 ± 11	76 ± 10	<0.001
Current smokers, n (%)		61 (34.1)	700 (45.2)	0.004
Hypertension, n (%)		144 (80.4)	955 (61.7)	<0.001
Creatinine, µmol/L		71.0 (61.8–83.2)	70.3 (62.3–79.3)	0.280
Heart failure, n (%)		27 (15.1)	96 (6.2)	<0.001
Elevated cardiac enzymes/markers, n (%)		139 (77.7)	1138 (73.6)	0.237
Cardiac arrest, n (%)		0 (0)	2 (0.1)	0.063
Diabetes, n (%)		120 (67)	677 (43.8)	<0.001
Dyslipidemia, n (%)		144 (80.4)	1240 (80.2)	0.926
Previous MI, n (%)		57 (31.8)	273 (17.6)	<0.001
Previous PCI, n (%)		64 (35.8)	277 (17.9)	<0.001
Family history of CAD, n (%)		96 (42.5)	477 (30.8)	0.002
GRACE risk				0.085
	Low	62 (34.8)	668 (43.2)	
	Intermediate	79 (44.4)	575 (37.2)	
	High	37 (20.8)	304 (19.7)	
Clinical diagnosis, n (%)		-	-	-
	UA	139 (77.7)	1138 (73.6)	0.237
	STEMI	16 (8.9)	208 (13.4)	0.089
	NSTEMI	24 (13.4)	201 (13)	0.876
Laboratory examinations				
	ALB (g/L)	41.17 ± 3.92	42.09 ± 3.65	0.002
	Lymphocyte count (×${\mathrm{10}}^{9}$/L)	1.66 (1.28–2.06)	1.74 (1.43–2.20)	0.007
	Neutrophil count (×${\mathrm{10}}^{9}$/L)	4.27 (3.29–5.20)	3.97 (3.19–4.90)	0.015
	Monocyte count (×${\mathrm{10}}^{9}$/L)	0.40 (0.30–0.50)	0.35 (0.29–0.45)	0.003
	hs-CRP	1.89 (0.74–5.16)	1.32 (0.63–3.36)	0.006
	TC (mmol/L)	4.15 ± 1.00	4.16 ± 0.95	0.432
	LDL-C (mmol/L)	2.46 ± 0.81	2.44 ± 0.81	0.817
	HDL-C (mmol/L)	1.04 ± 0.24	1.03 ± 0.23	0.700
	TG (mmol/L)	1.37 (0.97–2.01)	1.46 (1.02–2.07)	0.155
	FBG (mmol/L)	6.50 (5.66–7.93)	5.74 (5.21–6.83)	<0.001
	Glycosylated hemoglobin (%)	6.70 (6.00–7.70)	6.00 (5.50–7.00)	<0.001
	LVEF (%)	62 (57–61)	65 (60–68)	0.001
Angiographic findings and PCI				
	LM/multi-vessel disease, n (%)	30 (16.8)	86 (5.6)	<0.001
	Proximal LAD stenosis, n (%)	106 (59.2)	763 (49.3)	0.012
	Bifurcation or trifurcation lesions, n (%)	135 (75.4)	1195 (77.2)	0.582
	SYNTAX score	27.8 ± 11.7	20.6 ± 10.6	<0.001
	DES, n (%)	144 (80.4)	1273 (82.3)	0.543
	BRS, n (%)	4 (2.2)	93 (6)	0.038
	DCB, n (%)	26 (44.8)	85 (24.1)	0.001
	In-stent restenosis, n (%)	43 (24)	158 (10)	<0.001
	Complete revascularization, n (%)	79 (44.1)	978 (63.2)	<0.001
Discharge medications				
	Aspirin, n (%)	173 (96.6)	1537 (99.4)	<0.001
	Clopidogrel, n (%)	160 (89.4)	1424 (92)	0.220
	Ticagrelor, n (%)	19 (10.6)	123 (8)	0.220
	Statins, n (%)	177 (98.9)	1547 (100)	<0.001
	ACEI/ARBs, n (%)	115 (64.2)	720 (46.5)	<0.001
	β-blockers, n (%)	133 (74.3)	1077 (69.6)	0.195
	Any antidiabetic treatment, n (%)	106 (59.2)	470 (30.4)	<0.001
	Insulin, n (%)	66 (36.9)	206 (13.3)	<0.001

PAD, peripheral artery disease; BMI, body mass index; SBP, systolic blood pressure; DBP, diastolic blood pressure; MI, myocardial infarction; PCI, percutaneous coronary intervention; CAD, coronary artery disease; GRACE, global registry of acute coronary event; UA, unstable angina; STEMI, ST-segment elevation myocardial infarction; NSTEMI, non-ST-segment elevation myocardial infarction; ALB, albumin; hs-CRP, high-sensitivity C-reactive protein; TC, total cholesterol; LDL-C, low-density lipoprotein cholesterol; HDL-C, high-density lipoprotein cholesterol; TG, triglyceride; FBG, fasting blood glucose; LVEF, left ventricular ejection fraction; LM, left main artery; LAD, left anterior descending artery; SYNTAX, synergy between percutaneous coronary intervention with taxus and cardiac surgery; DES, drug eluting stent; BRS, bioresorbable scaffold; DCB, drug coated balloon; ACEI, angiotensin-converting enzyme inhibitor; ARBs, angiotensin receptor blockers.

### 3.2 PAD’s Predictive Value for the Primary Endpoint

Table 2 summarizes clinical outcomes in the PAD and non-PAD groups. After 1109 days of follow-up (IQR, 927 to 1109 days), 354 individuals showed at least one primary endpoint’s component, including 44 (2.5%) all-cause deaths, 37 (2.1%) cardiovascular deaths, 47 (2.8%) MI cases, 24 (1.4%) stroke cases and 289 (16.7%) cases of unplanned repeat revascularization. The incidence rates of MACE (40.3% vs. 17.9%), all-cause death (11.2% vs. 1.6), cardiovascular death (8.9% vs. 1.4), myocardial MI (8.4% vs. 2.2) and unplanned repeat revascularization (30.2% vs. 15.2%) all showed marked elevations in the PAD group in comparison with non-PAD cases (*p* < 0.001).

**Table 2.** **Clinical outcomes during follow-up stratified by PAD status**.

	With PAD (n = 179)	Without PAD (n = 1547)	*p* value
MACEs, n (%)	77 (43.0)	277 (17.9)	<0.001
All-cause death, n (%)	20 (11.2)	24 (1.6)	<0.001
Cardiovascular death, n (%)	16 (8.9)	21 (1.4)	<0.001
MI, n (%)	15 (8.4)	34 (2.2)	<0.001
Stroke, n (%)	10 (5.6)	14 (0.9)	<0.001
Unplanned repeat revascularization, n (%)	54 (30.2)	235 (15.2)	<0.001

PAD, peripheral artery disease; MACEs, major adverse cardiovascular events; MI, myocardial infarction.

**Fig. 1.**
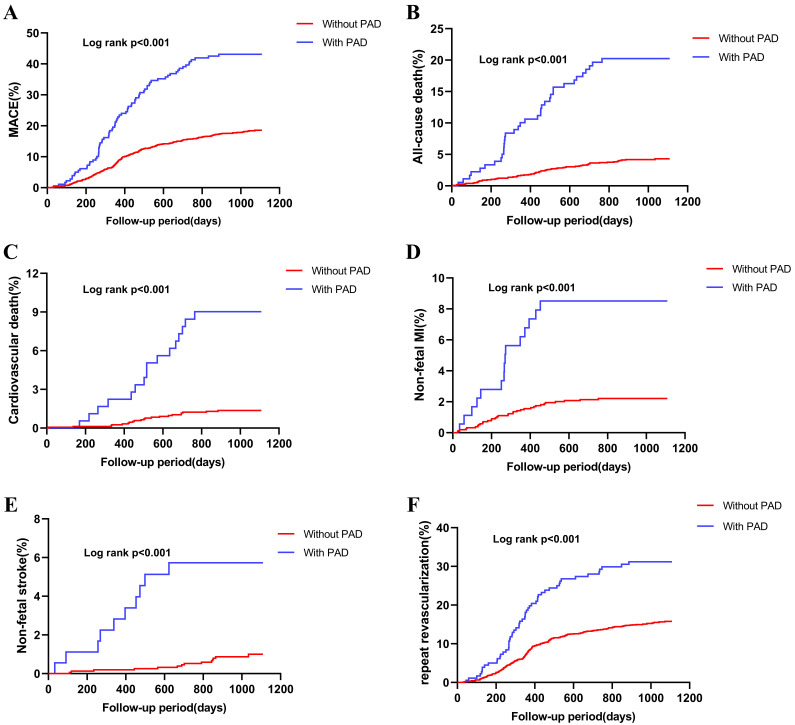
**Kaplan–Meier survival curves for MACE (A), all-cause death (B), cardiovascular death (C), MI (D), stroke (E), and repeat revascularization (F) analyses in the patients with or without PAD**. MACE, major adverse cardiovascular event; PAD, peripheral artery disease; MI, myocardial infarction.

Kaplan-Meier curves for the PAD and non-PAD populations showed significantly elevated rates of MACEs and individual events in PAD cases versus the non-PAD group (all log-rank *p* < 0.001; Fig. 1). Interestingly, PAD independently predicted MACEs even upon adjustment for other confounding variates (HR = 1.735, 95% CI: 1.281–2.351; Table 3).

**Table 3.** **Independent risk factors for MACEs in multivariate regression analysis**.

Variable		HR (95% CI)	*p*-value
PAD			
	Without PAD	ref	ref
	With PAD	1.735(1.281–2.351)	<0.001
Age		1.032 (1.015–1.049)	<0.001
Current smokers		1.486 (1.141–1.935)	0.003
Previous PCI history		1.465 (1.105–1.943)	0.008
Heart rate at admission		1.019 (1.007–1.032)	0.002
SBP		1.023 (1.015–1.031)	<0.001
DBP		0.967 (0.955–0.979)	<0.001
Killip classification		0.763 (0.612–0.952)	0.016
Cardiac arrest		4.934 (1.127–21.592)	0.034
FBG		1.187 (1.107–1.273)	<0.001
Complete revascularization		0.624 (0.487–0.799)	<0.001
Discharged with β-blockers		0.697 (0.552–0.879)	0.002

MACEs, major adverse cardiovascular events; HR, hazard ratio; CI, confidence interval; PAD, peripheral artery disease; PCI, percutaneous coronary intervention; SBP, systolic blood pressure; DBP, Diastolic blood pressure; FBG, fasting blood glucose.

**Fig. 2.**
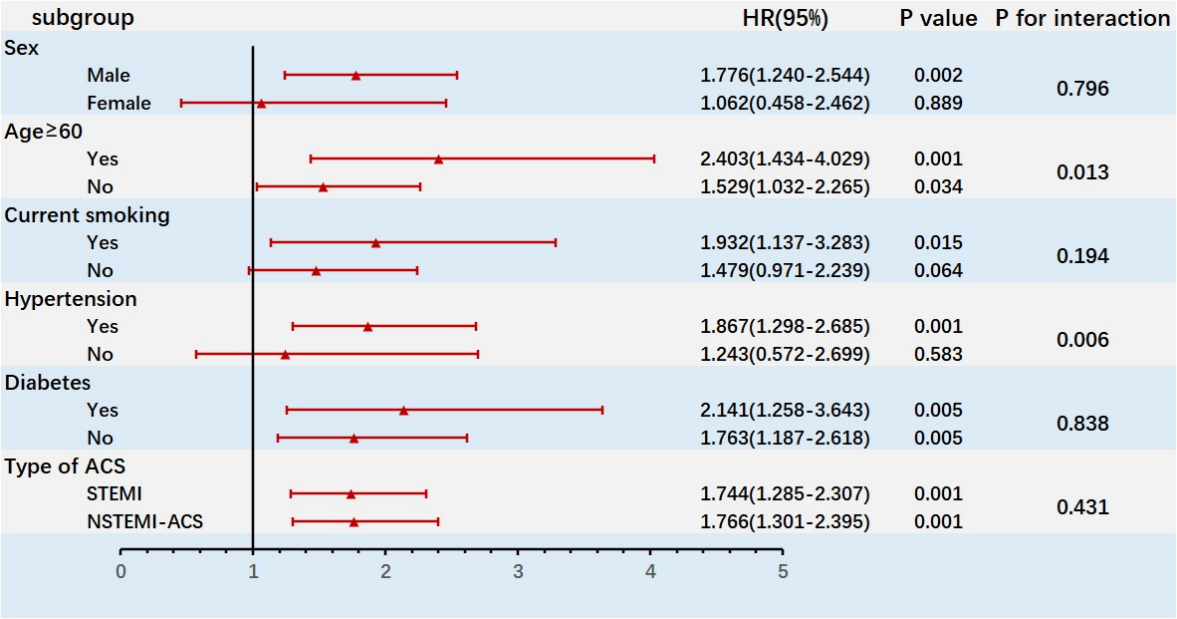
**Subgroup analysis of the effect of PAD on the risk of MACEs**. HR, hazard ratio; ACS, acute coronary syndrome; STEMI, ST-segment elevation myocardial infarction; NSTEMI, non-ST-segment elevation myocardial infarction; PAD, peripheral artery disease; MACEs, major adverse cardiovascular events.

A subgroup analysis was conducted to assess the differential effect of PAD on MACEs in various patient groups (Fig. 2). PAD’s predictive powers for MACEs were similar in subgroups based on gender, current smoking status, diabetes status, and STEMI or NSTE-ACS occurrence. However, PAD had significant interactions with age and hypertension. PAD exerted larger effects on MACE in older patients (interaction *p* = 0.013) and those with hypertension (interaction *p* = 0.006).

### 3.3 Incremental Effect of PAD on the Predictive Power of the Primary Endpoint

Next, we examined whether PAD ameliorates the capability of the GRACE scale in predicting MACE occurrence. As shown in Table 4, PAD addition resulted in markedly enhanced predictive ability for MACEs in comparison with the baseline GRACE score (Harrell’s C-index: GRACE score + PAD vs. GRACE score: 0.610 vs. 0.587, NRI = 0.134 and IDI = 0.035; all *p* < 0.001). Moreover, PAD addition remarkably improved the C-indexes of GRACE scores for death and death + stroke + MI (GRACE score + PAD vs. GRACE score: 0.664 vs. 0.632 [*p* = 0.006] and 0.644 vs. 0.617 [*p* = 0.019], respectively).

**Table 4.** **Model performance after addition of PAD to the baseline GRACE model**.

		C-Statistic (95% CI)	*p*-value	NRI (95% CI)	*p*-value	IDI (95% CI)	*p*-value
MACE							
	GRACE	0.587 (0.268–0.922)	Ref	Ref	Ref	Ref	Ref
	GRACE+PAD	0.610 (0.287–0.933)	<0.001	0.134 (0.060–0.199)	<0.001	0.035 (0.017–0.059)	<0.001
Death							
	GRACE	0.632 (0.600–0.767)	Ref	Ref	Ref	Ref	Ref
	GRACE+PAD	0.664 (0.601–0.724)	0.006	0.000 (–0.002–0.035)	0.249	0.096 (–0.179–0.199)	1.065
Death, stroke, or MI							
	GRACE	0.617 (0.591–0.708)	Ref	Ref	Ref	Ref	Ref
	GRACE+PAD	0.644 (0.593–0.712)	0.019	0.006 (0.000–0.039)	0.030	0.125 (–0.082–0.226)	0.249

PAD, peripheral artery disease; GRACE, global registry of acute coronary events; NRI, net reclassification improvement; IDI, integrated discrimination improvement; MACE, major adverse cardiovascular event; Ref, reference; MI, myocardial infarction.

## 4. Discussion

This study evaluated PAD’s predictive power for adverse outcomes in ACS cases after PCI. The results indicated that MACE incidence was markedly elevated in PAD cases in comparison with non-PAD cases. After adjustment for potential confounders, PAD remained strongly correlated with poor prognosis. Additionally, PAD addition markedly enhanced the predictive capability of the GRACE system for MACEs.

PAD has been detected in 5% to 20% of CAD cases [9, 10, 11, 12, 13, 14, 15]. In an early study including 10,440 patients undergoing PCI, symptomatic PAD was found in 18.9% of patients [9]. In another early trial reported by the Northern New England Cardiovascular Disease Study Group, 13.4% of individuals with multivessel disease administered either PCI or CABG were considered PAD cases [10]. However, PAD prevalence rates in CAD cases were much lower in several recent reports. In the multicenter e-ULTIMASTER registry including 37,198 PCI patients, PAD was found at 6.7% [11]. Another recent report based on a single-center prospective PCI registry including 25,690 PCI patients found that PAD occurred in 6.3% of the cases [12]. The falling trend of PAD was documented by a nationally representative study of 4.6 million individuals in the UK [16], which found an astonishing 15% reduction in the standardized PAD incidence between 2006 and 2015, possibly due to early identification and treatment. Of the ACS patients administered PCI in this study, 10.4% had PAD, which is within the previously reported range but higher than the prevalence rates in recent reports. A possible explanation for prevalence variability could be differences in study populations, regions, and diagnostic criteria. We only included ACS cases administered PCI, who had higher odds of having comorbidities compared with stable CAD patients. Other studies only examined PAD patients with a prior history or current symptoms [13, 14]. In contrast, we also included asymptomatic patients with positive ultrasound findings, who are easily missed but have similar risk profiles for morbidity and mortality as those with symptoms [17].

Consistent with previous studies [9, 10, 11, 12, 13, 14, 15], we found that PAD conferred higher risk profiles and worse clinical outcomes. PAD patients were older, had elevated prevalence rates of cardiovascular risk factors and higher SYNTAX scores, but lower odds of achieving complete revascularization. Meanwhile, the proportion of smoking is lower in PAD patients than that in those without PAD. The exact reason is not known. One possible reason is that only the information of current smoking was collected in our registry dataset. Most of PAD patients in our study were diagnosed previously. It is possible that a substantial number of smokers had quitted smoking after the diagnosis of PAD, and were not indicated as ‘current smoker’. Furthermore, in this study, the utilization rate of DCB was relatively high. This study is based on a cohort dataset which represent the real-world clinical situation. There are interventional cardiologists who prefer DCB to stenting under certain situations, especially for treatment of small vessels, in-stent restenosis or bifurcation lesions. The proportion of PCI with in-stent restenosis in PAD patients was significantly higher than that in patients without PAD (24.0% vs. 10.0%, *p* < 0.001, Table 1), which could partly explain the higher rate of DCB use in PAD patients.

Previous studies consistently indicated independent associations between PAD and enhanced risk of mortality and cardiovascular events in several clinical settings, including in patients with prior MI [18], undergoing PCI or CABG [9, 10, 11, 12, 13, 14, 15], or post-ACS [19, 20]. In a joint database of post-ACS patients in 4 trials, the incidence of MACEs in PAD cases was 1.6 fold that of those without PAD20. In recent reports assessing patients undergoing PCI, PAD was consistently reported to independently predict MACEs and death, with hazard ratios varying between 1.3 and 2.0 [9, 10, 11, 12, 13, 14]. In this work, only ACS cases administered PCI were included. Our results confirmed previous findings and demonstrated that PAD patients had markedly elevated incidence rates of MACEs and individual MACE components compared with those without PAD. The association between PAD and increased risk of MACE in ACS patients after PCI is complex. The worse clinical outcomes related to PAD could not be completely attributed to differences in risk profiles. After adjusting for all comorbidities and angiographic indexes, PAD still independently predicted a worse clinical outcome, which conferred a 73.5% increase in MACE risk. Patients with PAD are more likely to have older age, more concomitant disorders, more complex lesions, and higher SYNTAX score, which are all relevant to worse clinical outcomes. Another possible explanation is that the higher atherosclerotic burden of PAD patients may be relevant to those risk factors which are not routinely measured, such as genetic background, increased inflammatory state, or higher level of cytokines.

Surprisingly, few reports have considered PAD a risk factor in predictive models. In the Dual Antiplatelet Therapy (DAPT) trial, although PAD was predictive of both the expected decrease of ischaemic events as well as the expected elevation of bleeding events with continued thienopyridine therapy beyond 12 months after PCI, it was not included in the DAPT score because the strengths of the associations were similar for both endpoints [21]. The GRACE score based on clinical indexes at discharge constitutes a potent predictive factor of short-term and long-term prognosis following ACS [22, 23, 24] and provides a more accurate risk assessment for patients admitted and discharged from hospital with integral parameters including age, systolic blood pressure, pulse, blood creatinine, Killip classification at presentation, cardiac arrest at admission, elevated markers of myocardial necrosis and ST-segment changes. Although TIMI is simpler to calculate, its identification accuracy is not as good as that of the GRACE score. Studies in China have shown that the GRACE score has better predictive value than the TIMI score for the prognosis of patients with acute non-ST-segment elevation myocardial infarction [25]. However, not all the validated predictive factors decreasing prognosis are included in the GRACE scale. As shown above, PAD addition further improved the predictive capability of the GRACE system for MACEs, albeit the improvement was small in magnitude (C-statistic 0.610 vs. 0.587). It may not be easy to improve such models including potent indexes as the GRACE scale. To deal with this anomaly, IDI and NRI were devised to evaluate reclassification with new molecular markers or variables [26, 27]. In this study, a significant improvement in net reclassification was obtained by using these two matrices after PAD addition to the GRACE score. These findings suggest this novel risk-prediction model might have high accuracy in predicting the outcome of ACS patients undergoing PCI in clinical practice.

As PAD has relatively low diagnosis cost and is a modifiable disease, the current study highlights the potential significance of early identification and treatment in this specific population. Given that PAD increases the risk of cardiovascular events, whether routine extremity ultrasound screening in ACS patients could help detect asymptomatic PAD and improve prognosis warrants further investigation by randomized trials.

Unfortunately, this study had several limitations. Firstly, the current study was a small-sample single-center observational trial. Secondly, ultrasound was not routinely applied for all patients. Some patients without symptoms or a previous history might be missed. Thirdly, three quarters of the ACS patients included in this study were UA, which may not reflect well on patients with acute myocardial infarction. Finally, treatment strategies for PAD, including medications or peripheral revascularization, were not considered in the current analyses, which might affect prognosis in PAD patients.

## 5. Conclusions

In ACS cases administered PCI, PAD independently predicted worse clinical outcomes. PAD addition could improve the performance of the GRACE risk score for MACEs. Further investigations are warranted to clarify the underpinning mechanism and to develop potential therapeutic strategies that would minimize such risk.

## Data Availability

The datasets used in the current study are available from the corresponding author upon reasonable request.

## Abbreviations

ACS, acute coronary syndrome; CHD, coronary heart disease; GRACE, global registry of acute coronary events; PAD, peripheral artery disease; CAD, coronary artery disease; PCI, percutaneous coronary intervention; MACE, major adverse cardiovascular event; UA, unstable angina; NSTEMI, non-ST-segment elevation myocardial infarction; STEMI, ST-segment elevation myocardial infarction; CABG, coronary artery bypass grafting; FBG, fasting blood glucose; hs-CRP, high-sensitivity C-reactive protein; BP, blood pressure; T2DM, type 2 diabetes mellitus; TC, total cholesterol; LDL-C, low-density lipoprotein cholesterol; TG, triglyceride; HDL-C, high-density lipoprotein cholesterol; MI, myocardial infarction; HR, hazard ratio; CI, confidence interval; NRI, net reclassification improvement; IDI, integrated discrimination improvement; SYNTAX, synergy between percutaneous coronary intervention with taxus and cardiac surgery; ACEI, angiotensin-converting enzyme inhibitor; ARB, angiotensin receptor blocker.

## Supplementary Material

Supplementary material associated with this article can be found, in the online version, at https://doi.org/10.31083/j.rcm2411332.
