# Supplementary material for: Prognostic Significance of Peripheral Artery Disease in Patients with Acute Coronary Syndrome Undergoing Percutaneous Coronary Intervention
Source: Rev Cardiovasc Med. 2023 Nov 24;24(11):332. doi: 10.31083/j.rcm2411332 (PMC11272883; doi:10.31083/j.rcm2411332)
Supplement: Supplementary file 1 [file 2153-8174-24-11-332-s1.docx]

Supplementary Table 1. Diagnosis of PAD patients.

| History of peripheral vascular intervention | 32 |
| --- | --- |
| Previous ultrasound or computed tomography angiography (CTA) confirmed | 103 |
| Admission ultrasound confirmation | 44 |

PAD, peripheral artery disease.
